# Supplementary material for: Kinetics of Biodiesel Production from Microalgae Using Microbubble Interfacial Technology
Source: Bioengineering (Basel). 2022 Nov 29;9(12):739. doi: 10.3390/bioengineering9120739 (PMC9774469; doi:10.3390/bioengineering9120739)
Supplement: Supplementary file 1 [file bioengineering-09-00739-s001.zip › bioengineering-2012722-supplementary.pdf]

Kinetics of Biodiesel Production from Microalgae using Microbubble Interfacial technology

Fahed Javed[a], Muhammad Waqas Saif-ul-Allah[b], Faisal Ahmed[b], Naim Rashid[d], Arif Hussain[a], William B. Zimmerman[c], Fahad Rehman[a*]

[a] Microfluidics Research Group, Department of Chemical Engineering, COMSATS University Islamabad, Lahore Campus, Pakistan.

[b] Process and Energy Systems Engineering Center-PRESTIGE, Department of Chemical Engineering, COMSATS University Islamabad, Lahore Campus, Pakistan.

[c] Department of Chemical & Biological Engineering, The University of Sheffield, UK.

[d] Division of Sustainable Development, College of Science and Engineering, Hamad Bin Khalifa University, Qatar. Foundation, Doha Qatar.

*Corresponding Author

Email: frehman@cuilahore.edu.pk **Tel:** +92-42-111-001-007 **Fax:** +92-42-9203100

Tables:

Table S-I: The designed experiments and their response with predicted values of RSM and GRU

**Run**	**A:Molar ratio**	**B:Catalyst loading**	**C:Time**	**Conversion**	**Predicted values of GRU**	**Predicted values of RSM**
		g wt.% of MO	Min	%	%	%
1	25	2.5	10	69.27	69.271324	72.95
2	15	5	90	85.97	86.01162	91.19
3	5	2.5	90	86.02	86.06134	82.34
4	15	2.5	50	89.52	89.621094	88.68
5	15	2.5	50	90.72	90.734703	88.68
6	25	2.5	90	98.8	98.845497	95.86
7	15	5	10	68.69	68.720703	67.29
8	5	5	50	78.32	78.388245	76.78
9	15	2.5	50	89.26	89.325653	88.68
10	5	2.5	10	70.44	70.504852	73.38
11	25	5	50	92.1	92.14489	89.82
12	15	0	10	28.22	28.294582	23.00
13	25	0	50	29.54	29.582661	31.08
14	15	2.5	50	82.92	82.911377	88.68
15	5	0	50	28.74	28.793556	31.02
16	15	0	90	29.56	29.610783	30.96

Table S-II: Goodness-of-Fit summary generated through RSM

Source	Sequential p-value	Lack of Fit p-value	Adjusted R^2	Predicted R^2	
Linear	0.0083	0.0012	0.4865	0.2739	
2FI	0.9444	0.0006	0.3563	-0.4554	
Quadratic	**< 0.0001**	**0.1239**	**0.9644**	**0.8118**	**Suggested**
Cubic	0.1239		0.9832		Aliased

Table S-III: Statistical ANOVA analysis of current RSM model

Source	Sum of Squares	df	Mean Square	F-value	p-value	
**Model**	10226.95	9	1136.33	49.22	< 0.0001	Significant
A-Molar ratio	85.74	1	85.74	3.71	0.0953	
B-Catalyst loading	5461.17	1	5461.17	236.56	< 0.0001	
C-Time	507.69	1	507.69	21.99	0.0022	
AB	42.12	1	42.12	1.82	0.2188	
AC	48.65	1	48.65	2.11	0.1899	
BC	63.52	1	63.52	2.75	0.1411	
A^2	12.77	1	12.77	0.5530	0.4813	
B^2	3730.02	1	3730.02	161.58	< 0.0001	

C^2	141.95	1	141.95	6.15	0.0422	
**Residual**	161.60	7	23.09			
Lack of Fit	117.93	3	39.31	3.60	0.1239	Not significant
Pure Error	43.67	4	10.92			
**Cor Total**	10388.55	16				

Figures

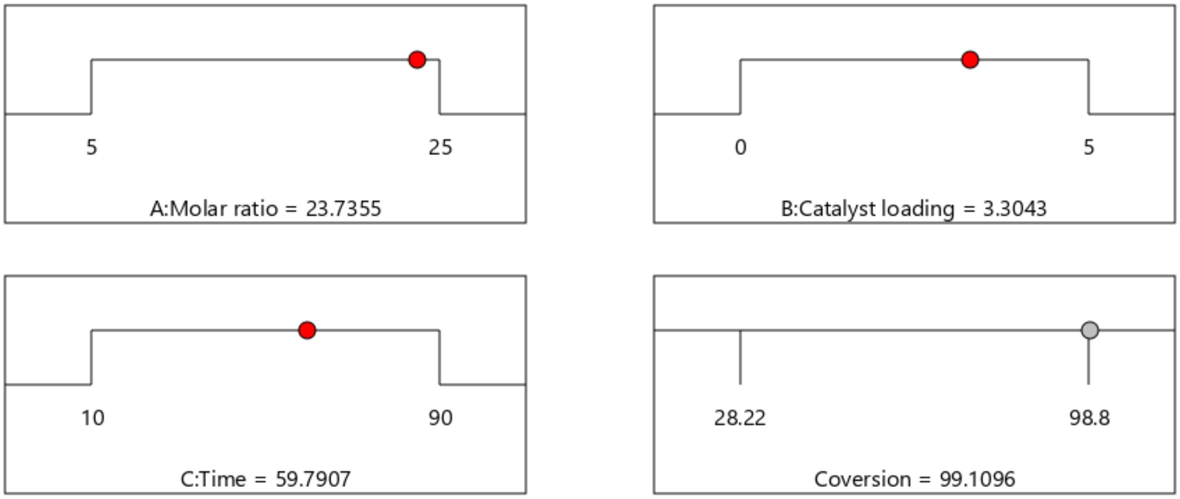

Figure S-I: Predicted values from RSM

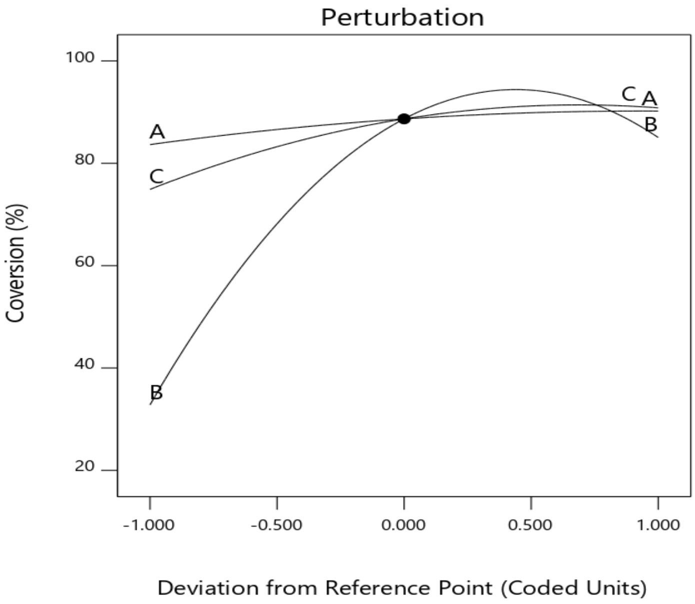

Figure S-II: Perturbation plot of experimental parameters

Statistical analysis through ANOVA

The goodness-of-fit summary provided by RSM shows that quadric model is best suited for current experimental design **Table S-II**. The suggested model is best suited for current experimental responses, and the current suggested model is also assessed through analysis of variance (ANOVA). The statistical analysis of ANOVA was shown in **Table S-III**. ANOVA gives a statistical analysis of the model and effects of different interactive parameters models [1]. The analysis shows a P-value less than 0.0001, which shows that the P-value is less than 0.05 indicating the design model is significant. In terms of F-value, the current model shows a larger F-value of 49.22 also indicates the model is significant. There is only a 0.01% chance of this higher F-value occurring due to noise.

In the current model, B, C, B^2, C^2 are significant model terms and values greater than 0.05 shows the model terms are not significant. The lack of fit F-value of the current model is 3.60, which indicates that lack of fits is not significant relative to the pure error. This not-significant value also implies that the quadric model is best suited for a current experimental response. [2]. The current quadratic model is further confirmed through correlation coefficient (R^2), adjusted $R^{2,}$ and predicted R^2. R^2 value indicates the relationship between predicted data by model and actual data, and near to 1 shows the significance between actual fit and predicted data [3]. In the current model, the value of R^2 to be 0.9844, predicted R^2 of 0.811 is in reasonable agreement with the adjusted R^2 of 0.9644. The difference of predicted R^2 and Adjusted R^2 is less than 0.2, indicating that the model is significant for the current study.

Referece

[1] A.I. Khuri, S. Mukhopadhyay, Response surface methodology, Wiley Interdisciplinary Reviews: Computational Statistics, 2 (2010) 128-149.

[2] A. Hosseinzadeh, A.A. Najafpoor, A.J. Jafari, R.K. Jazani, M. Baziar, H. Bargozin, F.G. Piranloo, Application of response surface methodology and artificial neural network modeling to assess non-thermal plasma efficiency in simultaneous removal of BTEX from waste gases: Effect of operating parameters and prediction performance, Process Safety and Environmental Protection, 119 (2018) 261-270.

[3] K. Carley, N. Kamneva, J. Reminga, Response Surface Methodology CASOS—Center for Computational Analysis of Social and Organizational Systems, in, Technical Report, ISRI—Institute for Software Research International …, 2004.
